# Changes in ventilation distribution during general anesthesia measured with EIT in mechanically ventilated small children

**DOI:** 10.1186/s12871-023-02079-z

**Published:** 2023-04-12

**Authors:** Dorothea Clasen, Isabel Winter, Stephan Rietzler, Gerhard K. Wolf

**Affiliations:** 1 Children’s Hospital Traunstein, Academic Teaching Hospital of Ludwig-Maximilians University Munich, Cuno-Niggl-Straße 3, 83278 Traunstein, Germany; 2 Dipl. Physicist Stephan Rietzler, Alpenstraße 17, 87734 Benningen, Germany

**Keywords:** General anesthesia, Ventilation distribution, Pediatrics, EIT, GI, ARDS

## Abstract

**Background:**

Atelectasis during general anesthesia is a risk for perioperative complications. EIT measurements were performed in mechanically ventilated healthy children during elective surgery to demonstrate the changes in ventilation distribution during general anesthesia. The ventilation distribution was quantified by calculating the Global Inhomogeneity index (GI).

**Methods:**

EIT measurements were performed in 23 children (9 weeks—10 years) without lung disease to detect changes in regional ventilation during elective surgery. Three previously defined time points were marked during the measurement: after intubation and start of pressure-controlled ventilation (PCV), change to pressure support ventilation (PSV), and after extubation (spontaneous breathing—SB). Ventilation distribution based on regions of interest (ROI) and changes in end-expiratory volume (∆EELV) were collected at these time points and compared. The Global Inhomogeneity index was calculated at the beginning of pressure-controlled ventilation (PCV).

**Results:**

With increasing spontaneous breathing, dorsal recruitment of atelectasis occurred. The dorsal ventilation fraction increased over the time of general anesthesia with increasing spontaneous breathing, whereas the ventral fraction decreased relatively (Difference ± 5.5 percentage points respectively; 95% CI; 3.5—7.4; *p* < 0.001). With the onset of spontaneous breathing, there was a significant reduction in end-expiratory volume (Difference: 105 ml; 95% CI, 75–135; *p* < 0.001). The GI of the lung-healthy ventilated children is 47% (SD ± 4%).

**Conclusion:**

Controlled ventilation of healthy children resulted in increased ventilation of the ventral and collapse of the dorsal lung areas. Restart of spontaneous breathing after cessation of surgery resulted in an increase in ventilation in the dorsal with decrease in the ventral lung areas. By calculating the GI, representing the ratio of more to less ventilated lung areas, revealed the presumed homogeneous distribution of ventilation.

**Trial registration:**

ClinicalTrials.gov Registration ID: NCT04873999. First registration: 05/05/2021.

## Background

Ensuring optimal ventilation during invasive ventilation contributes to lung protection. High ventilation pressures, high tidal volumes and regional atelectasis formation contribute significantly to ventilation-induced lung injury [1]. Electrical impedance tomography (EIT) allows bedside and radiation-free imaging of ventilation in real time [2]. Barber and Brown described this technology as early as the mid-1980s [3]. Various studies have shown good correlation of EIT and CT images in the detection of regional lung changes [4–6]. It has already been shown in some studies that dorsal atelectasis and a reduction in functional residual capacity occur during mechanical ventilation in the context of general anesthesia in adults [7–10]. EIT measurements have also shown that significant overdistension of ventral lung areas occurs in pediatric ARDS patients in order to allow for complete recruitment of the dorsal atelectatic lung areas [11]. EIT-guided recruitment and PEEP setting is a suitable way to achieve the optimal ratio of overdistension to collapse [12–14].

There are already numerous publications for the use of EIT measurements in adults. Data also exist for larger children with a thoracic circumference greater than 70 cm. However, no pediatric EIT belts (thoracic circumference ˂ 70 cm) were commercially available for the PulmoVista EIT device from Dräger until 2021. Therefore, one objective of the current study was to collect data on infants, toddlers, and children with a chest circumference between 36 and 70 cm.

Gravity affects the ventilation distribution of ventilated lungs. The purpose of this study was to investigate changes in ventilation distribution during general anesthesia in lung-healthy ventilated children. The question is whether the influence of gravity can be detected by EIT, although the dead weight of the lungs is lower in children than in adults. Furthermore, this study aims to quantify the presumed homogeneity of the ventilation distribution in lung-healthy children by calculating the Global Inhomogeneity index (GI).

## Methods

### Subjects

The study protocol was approved by the ethics committee of the Ludwig-Maximilians University Munich. This sample size was chosen in order to additionally perform a correlation analysis of the impedance and volume curves. A sample size of 19 participants was calculated. Taking into account 15% possible dropouts, 23 patients were included. Written informed consent was obtained from the parents or legal guardians before participation in the study. Pediatric patients receiving balanced general anesthesia (for induction: intravenous application of propofol 1% and sufentanil or alfentanil, for maintenance: inhalation application of sevoflurane) without using neuromuscular blockers as part of elective surgery, with a chest circumference between 36 and 70 cm and a tidal volume > 20 ml were included. A PEEP of 5 mbar was chosen for all patients. The ventilation pressure was chosen to achieve a tidal volume of 6 ml/kg/body weight. The highest driving pressure required was 11 mbar, the lowest 5 mbar. Patients with a permanent or temporary pacemaker, defibrillator or other implanted medical devices that deliver electrical energy were excluded. Patients in whom the application of the electrode belt could have been affected by wound care or infections in the chest area and female patients in whom pregnancy could not be excluded were not included. Measurements were taken during minor procedures such as closure of inguinal hernias, circumcision, orchidopexy or removal of dermoid cysts. The duration of sedation was shorter than one hour in all patients.

### Setting

Elective pediatric surgical operations at the Traunstein Hospital, Academic Teaching Hospital of the Ludwig Maximilian University of Munich, Germany.

### Cardiopulmonary monitoring

Vital signs, transcutaneous oxygen saturation (SpO_2_) and respiratory variables were monitored continuously and documented every 5 min. Ventilation parameters (end-tidal CO_2_ concentration, inspiratory and expiratory tidal volumes) were monitored by the anesthesia machine (Primus, Dräger Medical Deutschland GmbH, Lübeck, Germany).

### Electrical impedance tomography

EIT measurements were performed with the Dräger PulmoVista 500 (Dräger Medical Deutschland GmbH, Lübeck, Germany). The 16-electrode belt was placed between the 3rd and 5th intercostal spaces. Ventilation data were continuously recorded by the EIT device via a serial interface (Medibus, Dräger Medical Deutschland GmbH, Lübeck, Germany).

The following EIT parameters were stored during the measurement phases: EIT tidal images, global impedance curves, regional impedance curves and tidal variation of regions of interest (ROI) 1–4, change in end-expiratory lung impedance (ΔEELI) and ventilator flow curve.

### Experimental protocol

Pediatric patients were screened for inclusion and exclusion criteria during the anesthesiology or pediatric surgery informed consent interview. Written informed consent was obtained from the parents/legal guardians after they were informed of the planned study. Demographic data, vital signs and medical history were obtained and documented.

For later data evaluation, three time points were defined, which were marked during data recording by means of event markers.

PCV = controlled ventilation, PSV = assisted ventilation, and SB = spontaneous breathing.

On the day of the intervention, the inclusion and exclusion criteria were reevaluated. General anesthesia was induced and maintained by the responsible anesthesiologist. The appropriate electrode belt was applied and the exact position on the thorax.

After a stable EIT signal could be derived, data recording was started before the operation began. To avoid artifacts and ensure usable data, no infant manipulations or ventilatory changes were performed for two minutes. All artifacts (e.g. due to cauterization) were marked during the further course of the measurement. After completion of surgery, extubation and sufficient spontaneous breathing, another two-minute rest measurement was taken.

The collected data were processed after measurement using the Dräger EIT Application Version 1.30 (Dräger Medical Deutschland GmbH, Lübeck, Germany).

### Global and regional tidal variation

Global ventilation was divided among the four horizontal regions and the percentage of ventilation was shown for each region. ROI were designated from ventral (ROI 1) to dorsal (ROI 4). The mean values of a one-minute time interval of the regional tidal variations at the previously defined time points (PCV, PSV, SB) were evaluated.

### End-expiratory lung impedance (EELI)

The difference between two end-expiratory images at two freely selectable cursor positions C1 and C2 quantifies the global and regional changes in end-expiratory lung impedance (∆EELI). This impedance change is shown on the one hand by colors and on the other hand by numerical parameters. The global numerical value represents variations in global end-expiratory status at cursor positions C1 and C2 relative to the global tidal variation at C1. Regional deviations within the defined ROI are represented by the value ∆EELI ROI [15].

The ∆EELI values were collected for the ventilation mode transitions PCV to PSV, PSV to SB and PCV to SB and the ∆EELV values were calculated. For this purpose, the ∆EELI value was multiplied by the measured tidal volume at the reference time (C1) (∆EELV = ∆EELI * TV _global ref C1_).

### Global Inhomogeneity index

One-minute sequences of each EIT data were selected to calculate the GI. The minute containing the event marker "PCV" was selected. The GI was calculated as described by Zhao [16] using the Matlab program EITdiag version 1.6. Using the EIT images, we first estimated the total lung area [17], then the actual ventilated areas were determined and cardiac-related artifacts were subtracted. The calculation is based on the sum of the absolute differences between the median impedance values of all pixels and each pixel value of the tidal image. The GI is considered an indicator of the distribution of ventilation area throughout the lungs. Comparability between patients is enabled by the quotient of the described sum and all pixel values of the calculated lung area [16].

The increasing inhomogeneous distribution of ventilation during controlled ventilation can be visualized with the GI. It quantifies the gas distribution in the lungs and thus enables good comparability. In healthy individuals with normal ventilation distribution, the quotient is 50%. In pathologically altered lungs, where there is unilateral pulmonary ventilation, the GI is 100% [16].

### Statistical analysis

The results of this publication are based on an exploratory analysis of the results of the data from ventilated children.

**Fig. 1:**
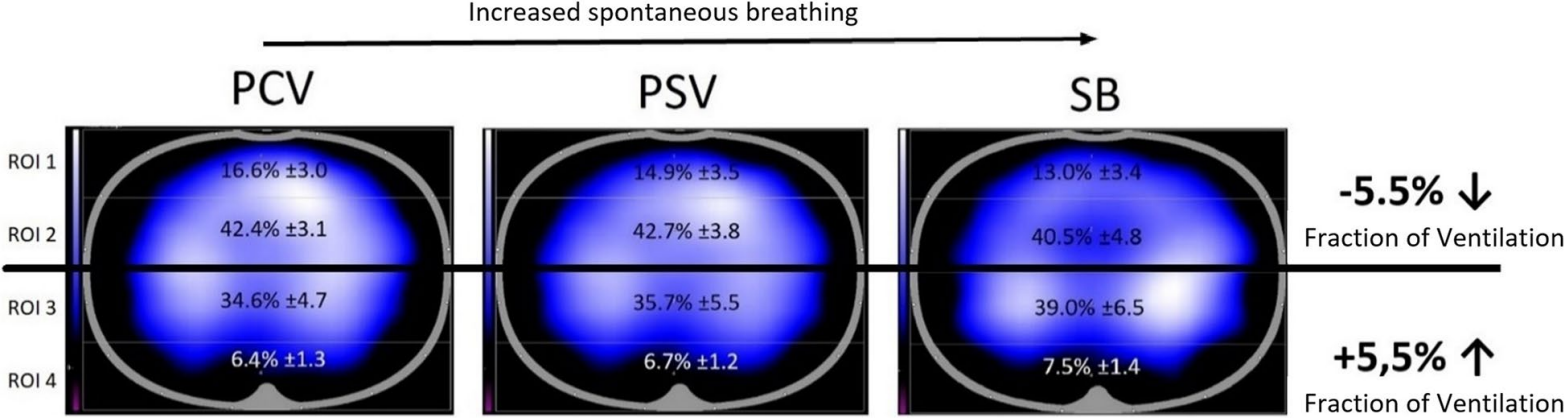
Ventilation distribution based on horizontal layers—ROI 1–4. The middle horizontal marker illustrates the grouping of ROI 1 and 2 as the ventral layer, and ROI 3 and 4 as the dorsal layer. The dorsal layer is 5.5 percentage points more ventilated at the time of spontaneous breathing (SB) than at the time of controlled ventilation (PCV)

**Fig. 2:**
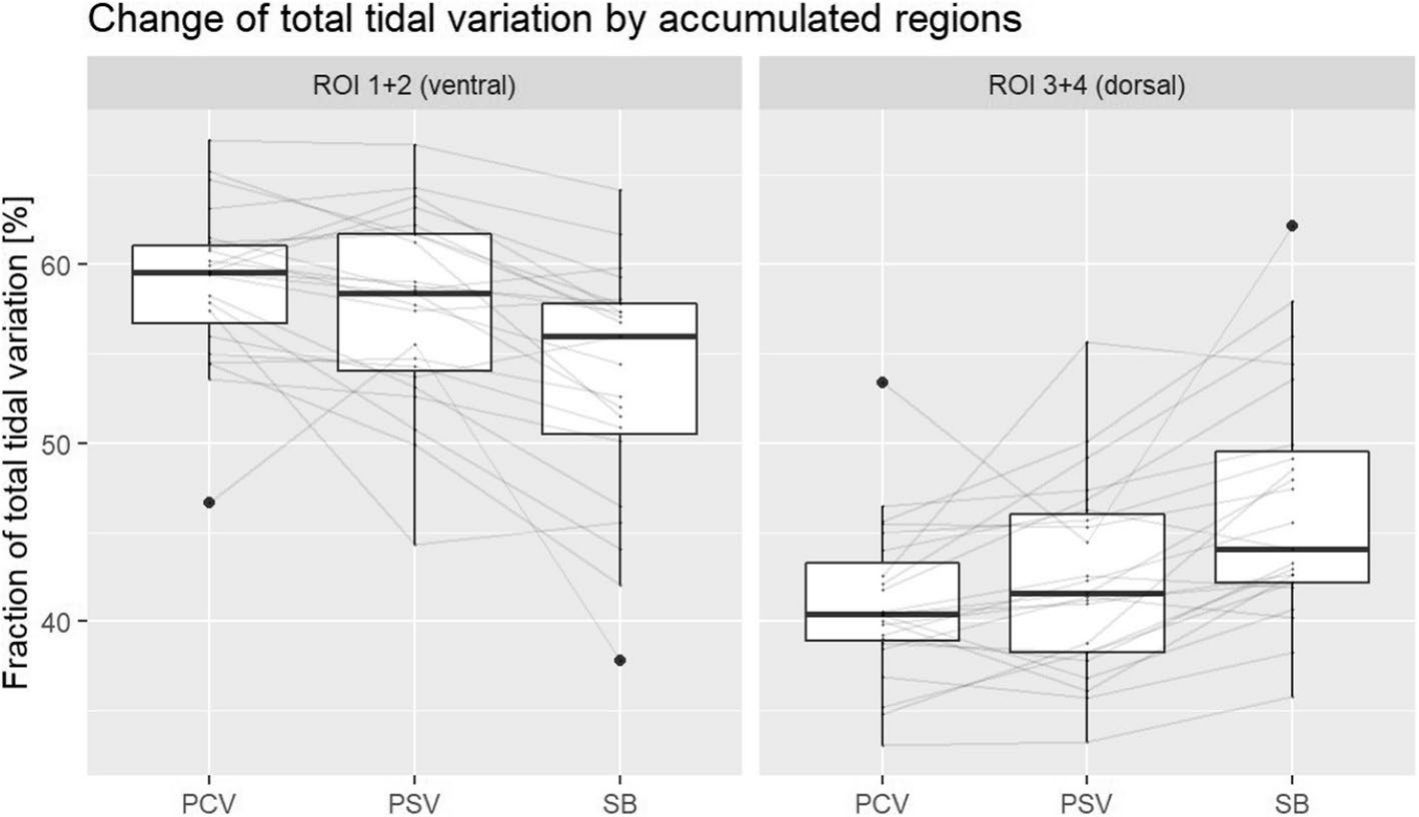
Plot of ventilation at the three intervention time points PCV, PSV and SB. ROIs 1 and 2 were combined into ROI ventrally, ROIs 3 and 4 into ROI dorsally. In the dorsal regions, there is an increase in ventilation by recruitment of dorsal atelectasis by a mean of 5.5 percentage points with increasing spontaneous breathing. In turn, the ventilation fraction of the ventral lung regions decreases

The change in regional ventilation fraction during general anesthesia was examined in an exploratory analysis. The changes were analyzed graphically (see Figs. 1 and 2) and tested for significance using a Wilcoxon signed-rank test.

**Fig. 3:**
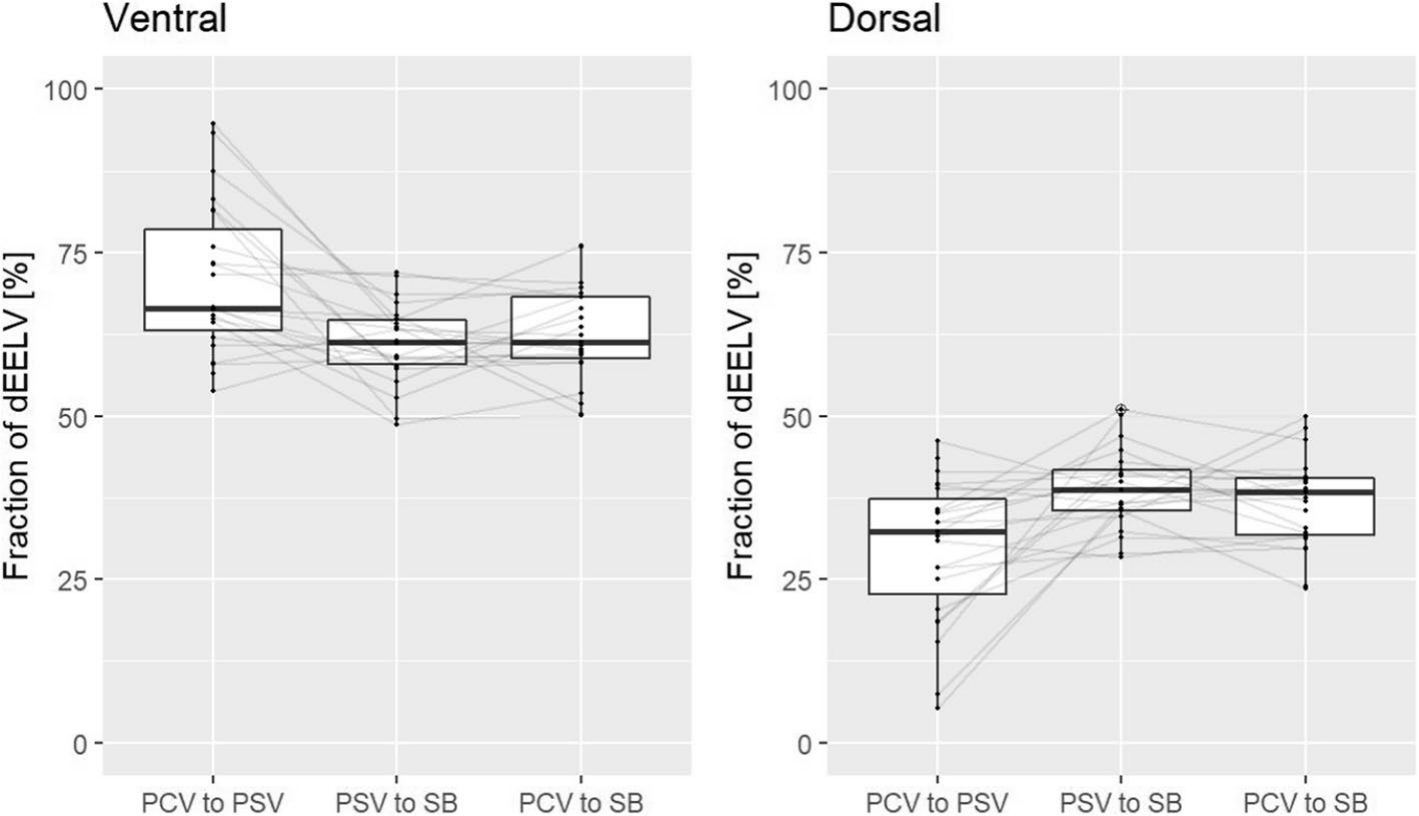
Plot of EELV changes at the three ventilation mode transitions. Ventral lung areas (left) and dorsal lung areas (right) show how the total ∆EELV change is proportionally distributed between the two regions at the corresponding ventilation mode transitions of ventilation. In addition to the boxplots, the change for each individual patient is plotted. To allow assignment over the course of individual study participants, the values are connected by gray lines. The values of the study population do not show a normal distribution. Due to statistical outliers, the median values are used as measure of location. The dorsal regions show an increase, which can be explained by the recruitment of atelectasis due to the onset of spontaneous breathing. Due to the loss of PEEP after termination of mechanical ventilation, the overdistension in the ventral lung regions decreases and thus the proportion of ∆EELV

A Wilcoxon signed-rank test was used to compare the reduction in EELV and the distribution of EELV during ventilation and spontaneous breathing (see Fig. 3).

All statistical analyses of this study were performed using the program "R" (version 4.2) and a *p*-value ≤ 0.05 was defined as statistically significant.

## Results

### Demographic data

Twenty-three patients were included in the study. All underwent EIT measurements during general anesthesia in the course of elective surgery. Demographic data of all study participants are shown in Table 1.

**Table 1:** Demographic data of healthy children

		**Overall, *N* = 23**	
**Age [years]**			
Mean (Range)		4.5 (0.2 – 10.0)	
**Height [cm]**			
Mean (Range)		109 (62—151)	
**Weight [kg]**			
Mean (Range)		20 (6—46)	
**Chest circumference [cm]**			
Mean (Range)		56 (38 – 71)	
**Belt Size**			
XS, *N* = 7	2XS, *N* = 11	3XS, *N* = 3	4XS, *N* = 2

#### Changes in regional ventilation during general anesthesia

Analysis of the changes in regional ventilation during general anesthesia in healthy children showed that with increasing efforts of spontaneous breathing, from PCV over PSV to SB, the proportion of ventilation in ventral areas decreased by 5.5 percentage points, whereas regional proportion recruitment occurred in dorsal areas. Throughout the measurement period, the majority of ventilation occurred in mid regions 2 and 3. As expected, region 4 showed the smallest proportion of ventilation (Fig. 1). When comparing mechanical ventilation and spontaneous breathing (PCV and SB as well as PSV and SB), significant changes in ventilation distribution were measurable in all four regions.

During controlled ventilation, more ventral lung areas were ventilated. The change of the ventilation mode from controlled to pressure-supported had a small and only in ROI 1 significant influence on changes in ventilation distribution (Difference ROI 1: 1.7 percentage points; 95% CI, 0.59—2.8; *p* = 0.003).

Dorsal ROI 3 was ventilated 4.4 percentage points (95% CI, -6.2—-2.8; *p* < 0.001) less during controlled ventilation compared to spontaneous ventilation. In contrast, ventral ROI 1 was ventilated 3.6 percentage points more (95% CI, 2.4—4.7; *p* < 0.001) and ventral ROI 2 was ventilated 1.9 percentage points more (95% CI, 0.35—3.5; *p* = 0.08).

During pressure-supported ventilation, dorsal ROI 3 was ventilated 3.3 percentage points (CI 95%; -5.0—-1.7; *p* < 0.001) less than during spontaneous breathing. The increase was also less pronounced in ROI 1 by 1.9 percentage points (CI 95%; 0.77—2.9; *p* = 0.003).

The ventilation fraction of dorsal ROI 4 was very low, therefore the ventral two regions (ROI 1 and 2) and the dorsal two regions (ROI 3 and 4) were combined. Figure 2 illustrates the increase of the dorsal ventilation fraction at the expense of the ventral one. With the onset of spontaneous breathing there was a significant increase of 5.5 percentage points (95% CI, 3.5—7.4; *p* < 0.001) in the dorsal ventilation fraction compared to pressure-controlled ventilation.

#### Changes in the ∆EELI/∆EELV

At all ventilation mode transitions a reduction in the end-expiratory volume was measured. With increasing spontaneous breathing the dorsal regions showed an increase, while the ventral regions decrease. Changing the mode of ventilation showed a very small EELV reduction, while the onset of spontaneous breathing showed a more pronounced reduction. Stopping mechanical ventilation with the onset of spontaneous breathing resulted in a significant reduction in EELV compared with the change in mode of ventilation (PCV to PSV: -37 ml [Range: -163—33 ml] vs. PSV to SB: -142 ml [Range: -384—-19 ml]; Difference: 105 ml; 95% CI 75–135; *p* < 0.001). Range is included because lung volumes are dependent on body size and therefore there are large differences in measured changes in EELV.

**Fig. 4:**
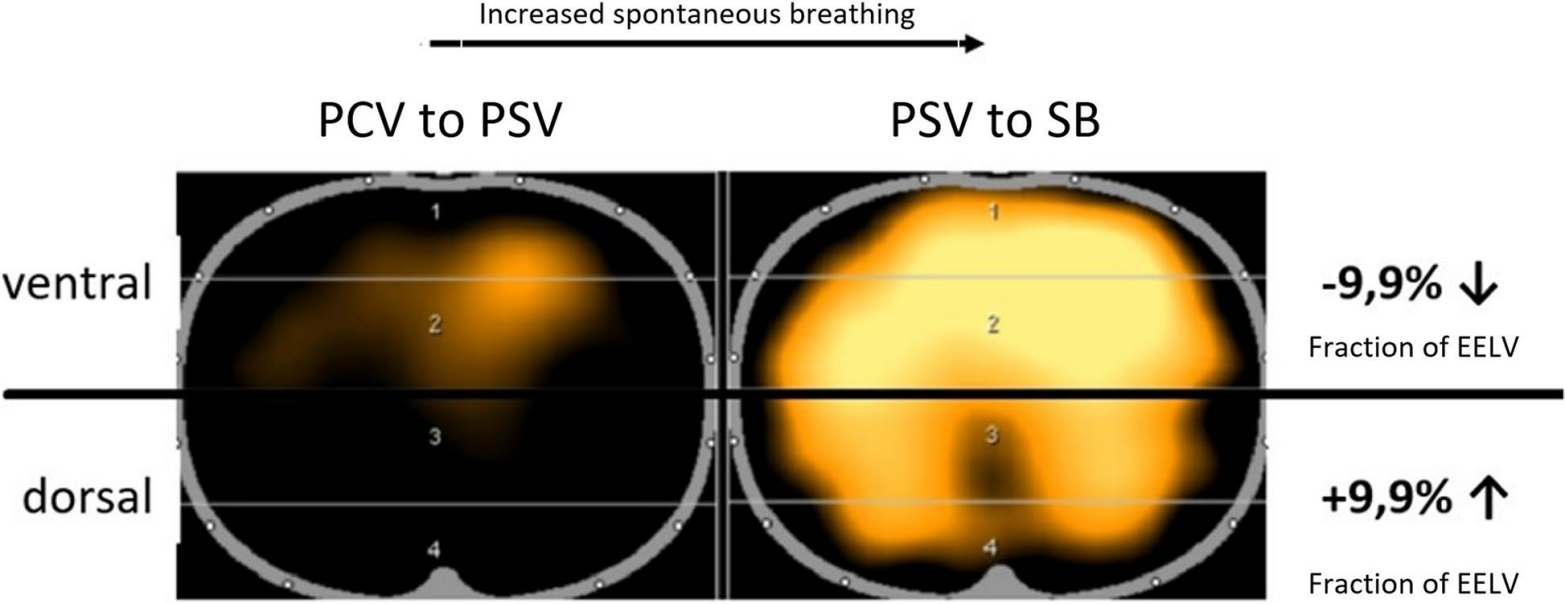
Exemplary representation of the EELV reduction at the different ventilation mode transitions. Orange: reduction in EELV; blue: increase in EELV; black: no change. Changing the mode of ventilation from PCV to PSV (left) shows only a small reduction of EELV which is mostly distributed in the ventral lung areas. After termination of mechanical ventilation (PSV to SB, right), the reduction is much more pronounced and distributed more evenly in both lung regions. The ventral portion decreases in favor of the dorsal by 9.9 percentage points

The distribution of ∆EELV in the different lung areas is shown in Fig. 3. The ventral regions (ROI 1 and 2) and the dorsal regions (ROI 3 and 4) were combined. During ventilation (PCV to PSV), a reduction in ∆EELV occurred mainly in the ventral lung regions with a proportion of 70.7%. After termination of mechanical ventilation (PSV to SB), there was a significant change in this distribution. The ventral proportion was reduced in favor of the dorsal proportion by 9.9 percentage points (Difference: 9.9 percentage points; 95% CI; 4.9–16; *p* < 0.001). Figure 4 is an exemplary representation of the regional change in EELV in this study.

#### Ventilation distribution of healthy children based on Global Inhomogeneity index

**Fig. 5:**
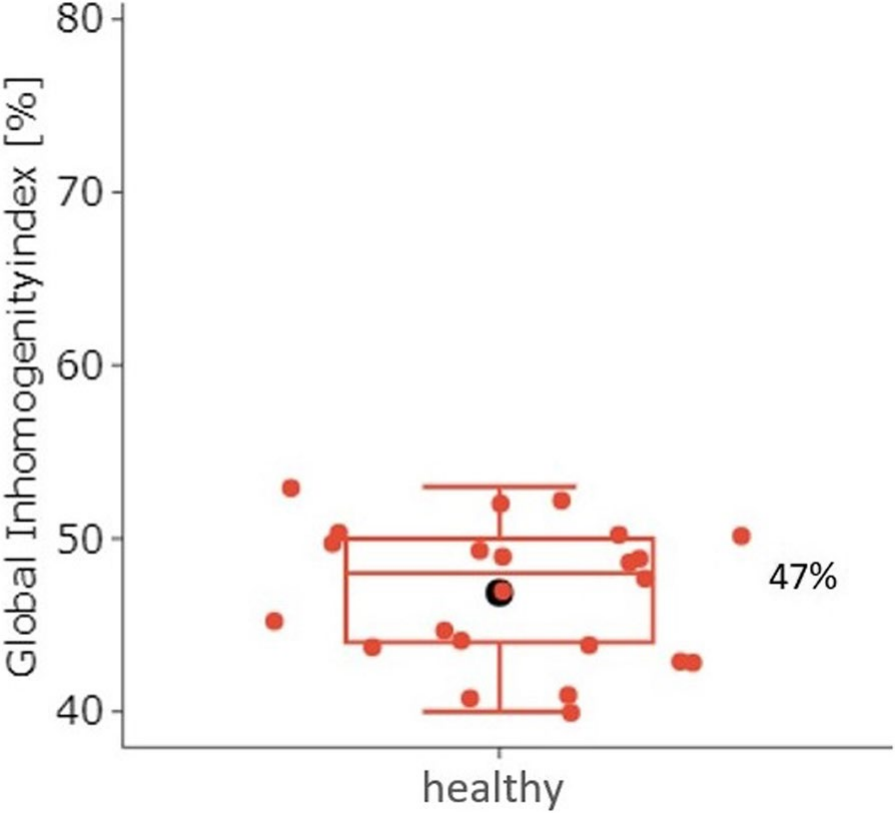
Global Inhomogeneity index (GI). The GI suggests that healthy children have a very homogeneous distribution of ventilation

The GI was calculated and showed a nearly homogeneous ventilation distribution with a GI of 47% (SD ± 4%, Range: 40, 53, Fig. 5).

## Discussion

The main findings of this study were: 1.) Dorsal proportion of ventilation occurring during general anesthesia was recruited with increasing spontaneous breathing, whereas the proportion of ventilation decreased in ventral regions. Changing the mode of ventilation from controlled to pressure-supported resulted in a significant reduction of ventilation only in the most ventral region. 2.) Spontaneous breathing after controlled ventilation was associated with a large reduction in end-expiratory volume due to termination of PEEP. 3.) The Global Inhomogeneity index showed homogeneous ventilation distribution of ventilated lung-healthy children.

Gunnarsson et al. [18] compared thoracic CT images of patients before and after induction of general anesthesia. In almost all patients, new onset of dorsal atelectasis was detected after induction of anesthesia. After induction of general anesthesia under controlled ventilation, relaxation of the diaphragm and a cranial shift occurs. This results in the formation of dorsal compression atelectasis [8, 9] and a reduction of the functional residual capacity [10].

In spontaneously breathing patients in the supine position, the dorsal portion of the diaphragm exhibits the greatest movement. Thus a thus a large proportion of ventilation occurs here. Positive pressure ventilation primarily moves the ventral regions of the diaphragm caudally. Therefore, during positive pressure ventilation, the majority of ventilation is distributed in the ventral lung areas [7].

In this study, this effect was investigated in infants and children. The performed exploratory analysis shows significant changes in ventilation distribution during general anesthesia. After induction of anesthesia and under controlled ventilation, the dorsal proportion of ventilation was low. This is probably due to a collapse of dorsal lung areas and increased ventral ventilation. During pressure-supported ventilation, there was already a minimal shift of ventilation towards the dorsal lung areas, although this difference was not significant in the current case series. This dorsal shift was most pronounced at the onset of spontaneous breathing after cessation of ventilation. There was a decrease in ventilation in the ventral areas, because the atelectatic lung areas were recruited. Further studies are needed, but this study provides evidence that controlled ventilation also leads to changes in ventilation distribution in infants, small and older children. Despite the lower weight, dorsal atelectasis with probable ventral lung overdistension may occur, among other reasons, due to the lack of diaphragmatic contraction.

During ventilation, positive end-expiratory pressure (PEEP) is applied. This pressure affects the end-expiratory volume of the lungs. As expected, a significant reduction in this volume was detected when comparing the two different modes of mechanical ventilation (with PEEP) and spontaneous breathing (without PEEP). While the change in ventilation mode only had a small effect on the change in end-expiratory lung volume, during spontaneous breathing, end-expiratory lung volume changed significantly and was distributed more evenly in both lung areas. The ventral portion became significantly smaller in favor of the dorsal portion. PEEP both recruited dorsal lung areas and overdistended ventral lung areas. After termination of mechanical ventilation, this PEEP ceased and a reduction in lung volume in these areas was detected. The dorsal tidal ventilation fraction was lower in this study compared with the ventral. Therefore, the dorsal fraction of the decrease in end-expiratory volume after extubation was also smaller than the ventral fraction. Wang et al. [19] performed perioperative EIT measurement in adults and also demonstrated a significant decrease in ∆EELI after extubation.

Since all children in this study had no pulmonary disease and were ventilated only for short periods, a roughly homogeneous ventilation distribution can be assumed. This could be confirmed by the GI calculation. The examined children showed an almost homogeneous ventilation distribution with a GI of 47%. Zhao et al. [20] compared the GI of adult healthy subjects and ARDS patients. The group of healthy patients had a significantly different ventilation distribution than the ARDS patients. The inhomogeneous distribution of ventilation in ARDS patients is rooted in infiltrates, edema, and the resulting atelectasis.

### Limitations

The sample size of this study is very small with 23 participants. In addition, there were large differences in age, height and weight of the patients. Therefore, the scatter of the data of the individual patients is sometimes very large and the difference of the mean values is small. The trend of changes of all patients in the same direction is present.

## Conclusions

This study shows that dorsal atelectasis with decrease in ventilation also occurs in infants, small and older children due to the influence of general anesthesia. At the same time, ventral lung areas are increasingly ventilated. With increasing spontaneous breathing, dorsal lung areas are recruited again. The Global Inhomogeneity index illustrates the homogeneity of the ventilation distribution in lung healthy children.

## Data Availability

The data sets generated and analysed in this study are not publicly available due to protection of minor patients and required consent by Dräger. They can be obtained from the corresponding author upon reasonable request.

## Abbreviations

ARDS: Acute respiratory distress syndrome
EELV: End-expiratory lung volume
EIT: Electrical impedance tomography
GI: Global Inhomogeneity index
PCV: Pressure controlled ventilation
PEEP: Positive endexpiratory pressure
PSV: Pressure support ventilation
ROI: Regions of Interest
SB: Spontaneous breathing
TV: Tidal Volume
